# Wheat Stripe Rust Grading by Deep Learning With Attention Mechanism and Images From Mobile Devices

**DOI:** 10.3389/fpls.2020.558126

**Published:** 2020-09-09

**Authors:** Zhiwen Mi, Xudong Zhang, Jinya Su, Dejun Han, Baofeng Su

**Affiliations:** ^1^College of Mechanical and Electronic Engineering, Northwest A&F University, Yangling, China; ^2^Key Laboratory of Agricultural Internet of Things, Ministry of Agriculture and Rural Affairs, Yangling, China; ^3^Shaanxi Key Laboratory of Agricultural Information Perception and Intelligent Services, Yangling, China; ^4^School of Computer Science and Electronic Engineering, University of Essex, Colchester, United Kingdom; ^5^State Key Laboratory of Crop Stress Biology for Arid Areas and College of Plant Protection, Northwest A&F University, Yangling, China

**Keywords:** wheat stripe rust, disease grading, CBAM module, C-DenseNet, attention mechanism

## Abstract

Wheat stripe rust is one of the main wheat diseases worldwide, which has significantly adverse effects on wheat yield and quality, posing serious threats on food security. Disease severity grading plays a paramount role in stripe rust disease management including breeding disease-resistant wheat varieties. Manual inspection is time-consuming, labor-intensive and prone to human errors, therefore, there is a clearly urgent need to develop more effective and efficient disease grading strategy by using automated approaches. However, the differences between wheat leaves of different levels of stripe rust infection are usually tiny and subtle, and, as a result, ordinary deep learning networks fail to achieve satisfying performance. By formulating this challenge as a fine-grained image classification problem, this study proposes a novel deep learning network C-DenseNet which embeds Convolutional Block Attention Module (CBAM) in the densely connected convolutional network (DenseNet). The performance of C-DenseNet and its variants is demonstrated *via* a newly collected wheat stripe rust grading dataset (WSRgrading dataset) at Northwest A&F University, Shaanxi Province, China, which contains a total of 5,242 wheat leaf images with 6 levels of stripe rust infection. The dataset was collected by using various mobile devices in the natural field condition. Comparative experiments show that C-DenseNet with a test accuracy of 97.99% outperforms the classical DenseNet (92.53%) and ResNet (73.43%). GradCAM++ network visualization also shows that C-DenseNet is able to pay more attention to the key areas in making the decision. It is concluded that C-DenseNet with an attention mechanism is suitable for wheat stripe rust disease grading in field conditions.

## Introduction

Stripe rust is a major wheat crop disease significantly affecting wheat yield and quality worldwide. With the continuous evolution and epidemic of new physiological races, and new pathogenic types of wheat stripe rust, the scope of the damage caused by wheat stripe rust has been further expanded recently, and the degree of damage has also become heavier. All these constraints have made stripe rust disease control more difficult. There are generally two ways available to control stripe rust for cereal crops including chemical control and genetic resistance (Ellis et al., 2014). Chemical control approach relies on the dynamic monitoring of pathogen populations, the prediction of disease outbreaks and the corresponding chemical intervention. While, genetic resistance approach mainly focuses on planting and distributing disease-resistant varieties, which is more cost-effective and sustainable in the long run (Li and Zeng, 2002).

**Figure 1:**
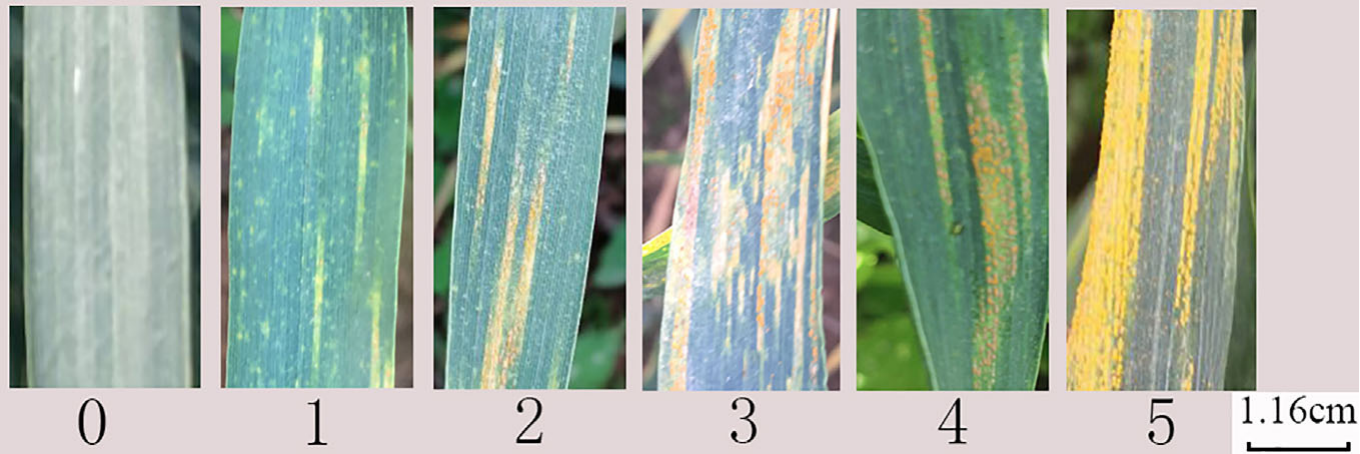
Example images for the identification standard in this work.

In disease-resistant wheat cultivar breeding, wheat condition is required to be monitored to make a preliminary judgment of its disease resistance. The disease resistance can be regarded as the expression of disease resistance genes and pathogenic bacteria genes (i.e., mutual gaming) in plants under the influence of certain environmental conditions. The long-term evolution process is that plants under different environmental conditions form different types of disease resistance. The criteria for dividing disease types are usually different. The main indicators for disease resistance performance of mature wheat plants are infection type and severity. In general, if the identification time is appropriate, the infection type is regarded as the disease resistance performance of the material itself, and the severity reflects whether this environment is conducive to sufficient disease incidence. Therefore, the infectious type is the main evaluation index for wheat resistance performance. Different grading standards for stripe rust disease infection are available in the literature such as 0-9 identification standard (Peterson et al., 1948; McNeal et al., 1971) and 0-5 identification standard (Li and Zeng, 2002). The latter is adopted in this study, since it is widely used in China where this research was conducted. Example images of the used standard are shown in **Figure 1**, and the specific division rules are given in **Table 1**.

**Table 1:** Classification standard for stripe rust infection type in this work.

Infection type	Symptom
0	No visible symptom
1	Necrotic areas without sporulation
2	Necrotic areas with restricted sporulation
3	Necrotic and chlorotic areas with small or medium size sporulation
4	Chlorotic areas with major sporulation
5	Abundant sporulation without chlorosis and necrosis

The current monitoring method is based on manual investigations by pathology experts, which is time-consuming, labor-intensive, and prone to errors depending on individual experience. With the advent of deep learning and computer vision in recent years, there is a trend to adopt these high-throughput information technology to detect and quantify wheat stripe rust disease more effectively and efficiently.

In recent years, great progress has been made in plant disease identification by using Deep Neural Networks (DNN). The PlantVillage initiative (Hughes and Salathé, 2015) is dedicated to generating plant leaf disease datasets, which have collected 50,000 images of healthy and infected leaves of 14 different crops with 26 different diseases. Mohanty et al. (2016) designed a disease recognition classifier using the above public dataset but only with a limited accuracy. The crop-conditional plant disease classification network proposed by Picon et al. (2019a) obtains a balanced accuracy of 98%, which incorporates the contextual information by concatenation at the embedding vector level. Ramcharan et al. (2019) trained a Convolution Neural Network (CNN) object detection model to recognize the foliar symptoms of diseases in cassava and deployed the model on mobile applications to test its performance on mobile images and videos. It is found that the performance on filed image and video is decreased in term of F-1 score. In particular, illumination, shooting angle and other factors affect the performance of the model, which also proves that the field image classification is very challenging. Picon et al. (2019b) and Lu et al. (2017) have made great progress in the classification of different wheat diseases. Although the network in the above study has achieved a higher accuracy in plant diseases classification, most of the disease classification objects are images with easily distinguishable color and shape features. Wheat stripe rust infection grading is, however, more difficult, since the differences in image features (e.g., color, shape) under different levels of stripe rust infection are usually tiny and subtle. Therefore, this challenge is formulated as a fine-grained image classification problem in this study.

Fine-grained image classification is a hot topic in the field of image classification. The positioning-classification method is usually effective. Early positioning-classification methods relied on strongly supervised learning and required a lot of manual work to label key areas of the image. For example, SP⁃DA-CNN proposed by Zhang et al. (2016) uses Part annotation in the CUB bird dataset to train the detection network and obtain hard attention corresponding to 7 different parts of the bird in the dataset. After the features are cut at the corresponding positions, they are used for image classification with better performance. The attention mechanism (Borji et al., 2013) does not rely on manual annotation and is an effective method with weakly supervised learning. In particular, the attention mechanism optimizes the model and makes more accurate judgments by assigning different weights to different parts of interest in the model and extracting more important and critical information therein. For example, Jaderberg et al. (2015) proposed a spatial transform network, which uses soft attention to sample on feature maps to obtain morphologically transformed features. Compared with the classical convolutional networks, it can extract spatial feature information more efficiently. The two-level attention model proposed by Xiao et al. (2015) applies both object-level and part-level attention, where convolutional networks are used to obtain objects level information and clustering method is adopted to get the key local area in order to use the multi-level information more accurately. Fu et al. (2017) combined visual attention with recursive structure, and fused features and attention weights at each level of the recursive network, thereby combining key region features at multiple scales in the model.

Inspired by the attention mechanism, this work proposes a novel deep learning network C-DenseNet for wheat stripe rust infection grading, where the developed C-DenseNet embeds Convolutional Block Attention Module (CBAM) in the densely connected convolutional network (DenseNet). The main contributions are summarized as below:

1. By formulating stripe rust disease grading as a fine-grained image classification problem, this paper proposes C-DenseNet to achieve adaptive calibration of feature channels and space.
2. An open-access wheat stripe rust grading dataset (WSRgrading dataset) is collected, which contain a total of 5,242 wheat leaves with 6 levels of wheat stripe rust infection. The dataset is collected in the field condition by various mobile devices and then manually calibrated according to the infectious classification standard.
3. Based on the above dataset, extensively comparative experiments (e.g., performance, attention visualization) are conducted on the C-DenseNet (and its variants) against the classical DenseNet and ResNet. It is shown that the proposed C-DenseNet outperforms the classical DenseNet and ResNet, showing the effectiveness of embedding attention mechanism.

## Materials and Methods

This section details the materials and methods adopted in this research, which include the collected image dataset, the proposed C-DenseNet and its variants and attention visualization.

### WSRgrading Image Dataset

The image dataset used in this research is introduced including image acquisition and image preprocessing.

#### Image Acquisition

In original experiment design (Wu et al., 2020), a total panel of over 1,500 wheat accessions were used to evaluate stripe rust responses and the wheat lines Avocet S, Mingxian 169, and Xiaoyan 22 were used as the susceptible controls. In this substudy, part of the wheat plots (with a number of about 200) were used for image collection, where adult-plant resistance evaluations were carried out at Yangling (34°17’ N, 108°04’ E, altitude 519 m) in Shaanxi province during the winter wheat cropping season (2018–2019). More details about plant growth, management, and evaluation time are available in the previous publication (Wu et al., 2020).

**Figure 2:**
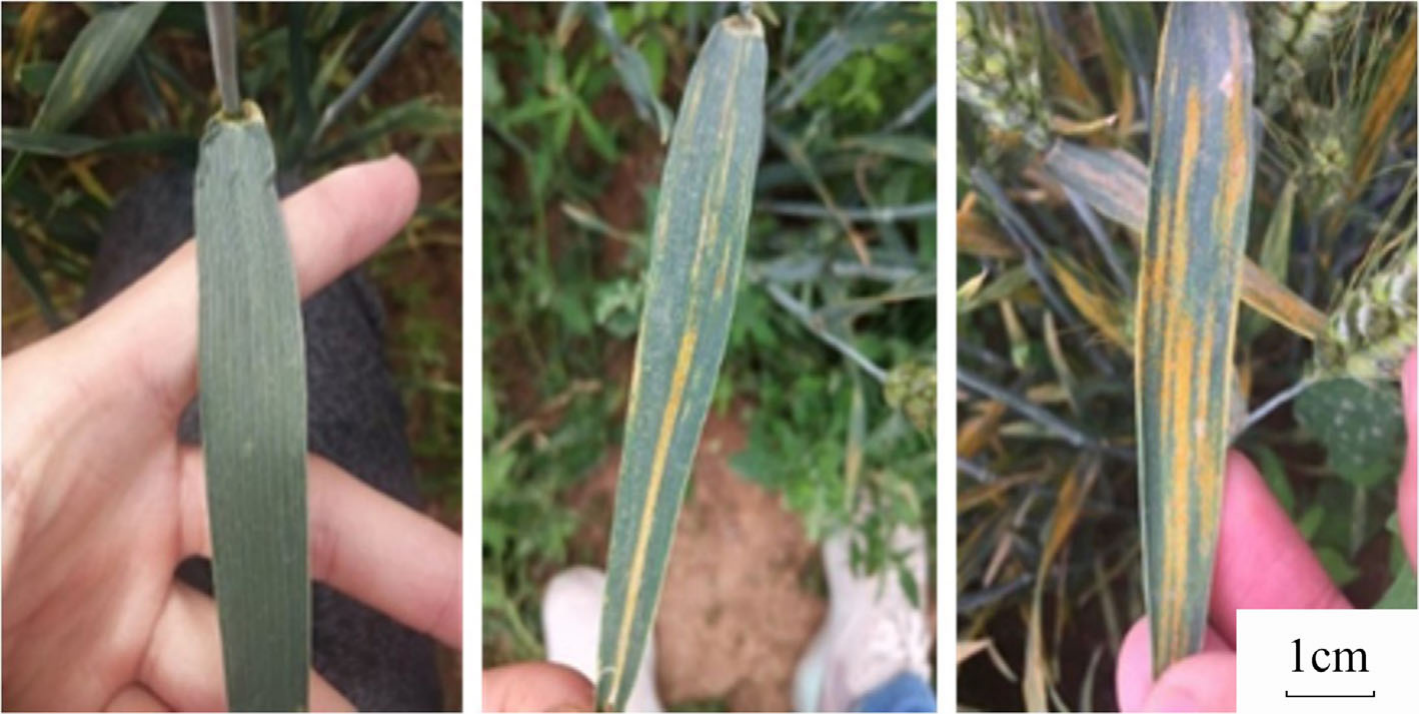
Example wheat leaves with strip rust infection in the dataset.

In this field, a total of 5,242 winter wheat leaf images (adult wheat plants after wheat earing) were collected by different mobile devices (e.g., Huawei Honor 9, Huawei Honor V9, Huawei Honor 10, vivo X20A, vivo X9Plus, Oppo R15x, Oppo A83T, Xiaomi Redmi 2 Pro, iPhone 6) with different imaging distances and angles, which were further divided into 6 levels of disease infection *via* visual inspection by pathology experts. Example image samples are shown in **Figure 2**. The dataset was collected between 8:00 AM and 5:00 PM covering various field conditions (e.g., illumination).

The images were acquired from the upper leaf surface and by avoiding direct sun light reflection. No other limitations were imposed on to maximally simulate the real acquisition conditions in real-life applications. The use of additional normalization color elements was also avoided as they are unpractical for field image acquisition as shown in Johannes et al. (2017).

A number of randomly selected samples are shown in **Figure 1**. It can be seen that the differences between wheat leaves with different levels of strip rust infection are usually tiny and subtle, which poses significant challenges for image classification. In this study, this challenge is formulated as fine-grained image classification problem. The number and proportion of images at each level are shown in **Table 2**. One can see from **Table 2** that the image numbers for different disease severity levels are generally balanced and therefore no data imbalance problem exists.

**Table 2:** Number and proportion of each category in the dataset.

Category	0	1	2	3	4	5
Number	661	980	961	895	738	1007
Proportion	0.126	0.186	0.183	0.171	0.141	0.193

#### Image Preprocessing and Augmentation

In the network proposed in this paper, the input image needs to be downsampled to an image of size 640 * 640. If a complex background is still left in the image, the classification performance of the developed model will be adversely affected. On the other hand, when the lesion is small, this direct downsampling method may make the lesion become very small or even disappear. In response to the above problems, we adopted a blade mask method similar to that proposed by Picon et al. (2019a).

**Figure 3:**
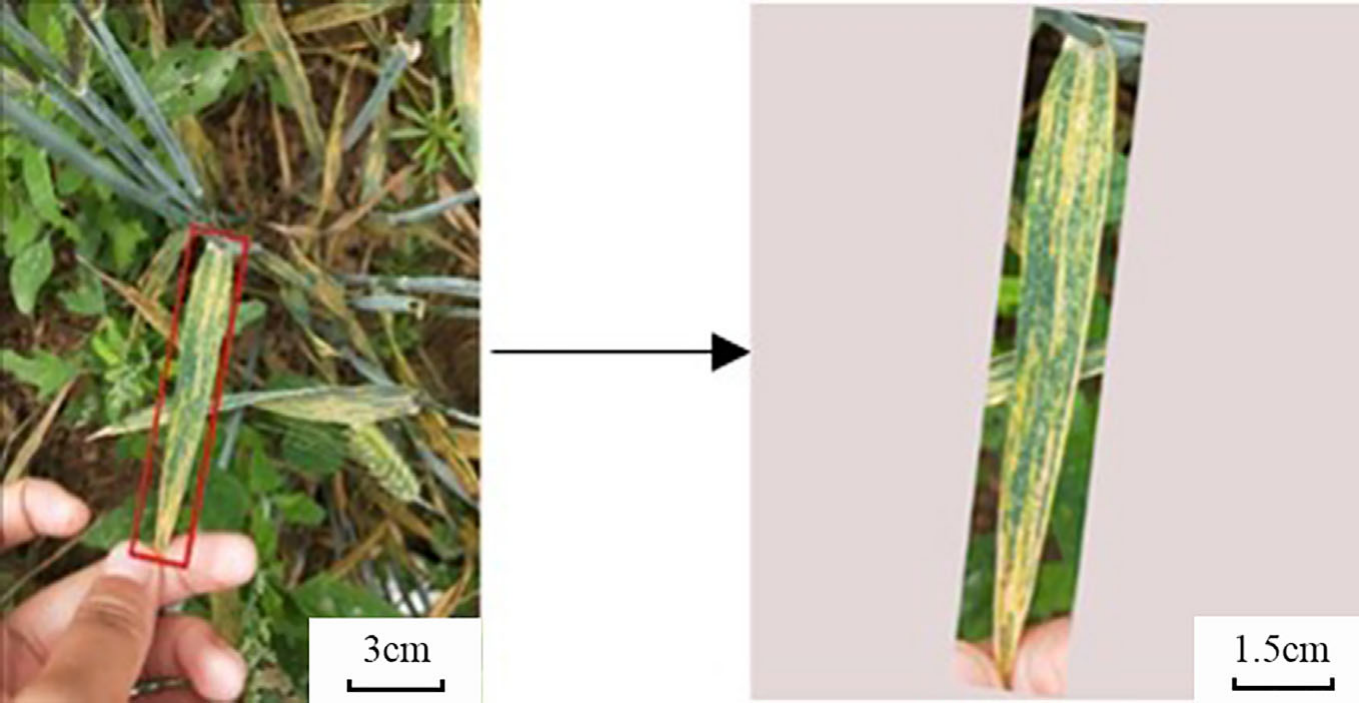
Sample images from the dataset after leaf mask-based cropping.

In this method, the image is cropped by a rotatable rectangular frame into a rectangular image containing leaf elements of interest. The range of leaf elements is provided by expert notes during the training phase and by the end-user during testing (or real-life applications). Then a white mask is adopted to expand the image into a square to avoid distortion when the image is normalized. Intuitively, cropping an image into a leaf boundary rectangle reduces the loss of detail by discarding non-relevant areas in the original image before downsampling, especially for early disease detection and grading. The details of this process are illustrated in **Figure 3**.

Enriching the dataset through data augmentation can increase data diversity and avoid overfitting problems in training the model. In order to increase the number and diversity of the original image dataset and make the extracted features more robust to changes in position and lighting, an enhanced image dataset was constructed by various data augmentation techniques including horizontal flipping, random angle rotation, and lighting changes (Dellana and Roy, 2016). In algorithm training phase, each image will be randomly transformed for geometric modification to ensure that the training image has a better variability. To avoid the problem of class imbalances during training (Japkowicz and Stephen, 2002), each class is also sampled uniformly from the data set, resulting in an equal percentage of each aggressive level class.

### C-DenseNet Network

DenseNet (Huang et al., 2017) is a densely connected network that implements feature multiplexing well. In DenseNet network, each layer obtains additional input from all the previous layers and passes its feature map to all subsequent layers in a cascade manner. Because each layer accepts feature mappings from all previous layers, the network can be thinner, more compact, and with fewer channels, which is a good departure from the single method of deepening the number of network layers and widening the network structure to improve network performance. It turns out that DenseNet performed better than Resnet (Chang et al., 2018) in actual training for stripe rust infection grading.

**Figure 4:**
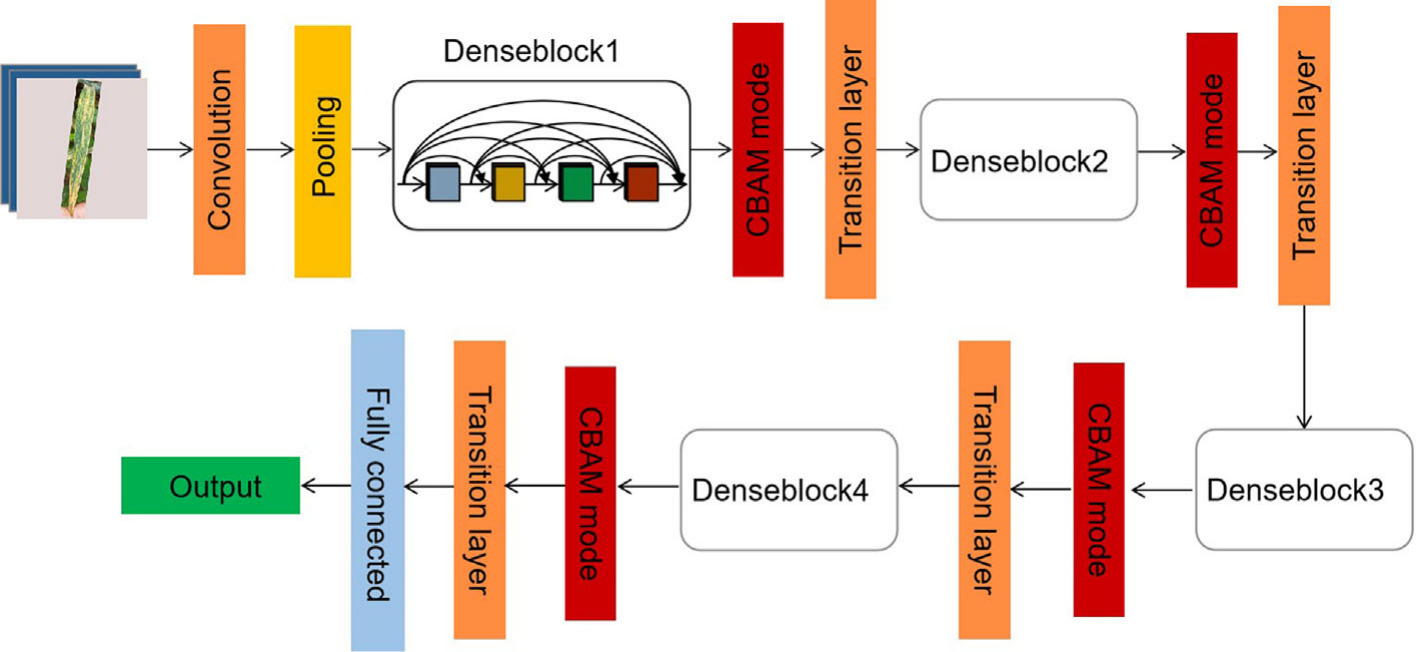
Framework of the proposed C-DenseNet for strip rust disease grading.

Although each infection level has different color and disease shape characteristics, the differences between some levels is very tiny and subtle. For example, the difference between the first and second levels is only the presence or absence of small spores. In addition, between second and fourth levels, third and fifth levels, the only difference is the presence or absence of white spots. To accurately identify these subtle differences, we designed the C-DenseNet network to implement the fine-grained image classification problem, as shown in **Figure 4**. The network is mainly composed of four dense blocks, three transition layers and three CBAM modules (Woo et al., 2018).

**Figure 5:**
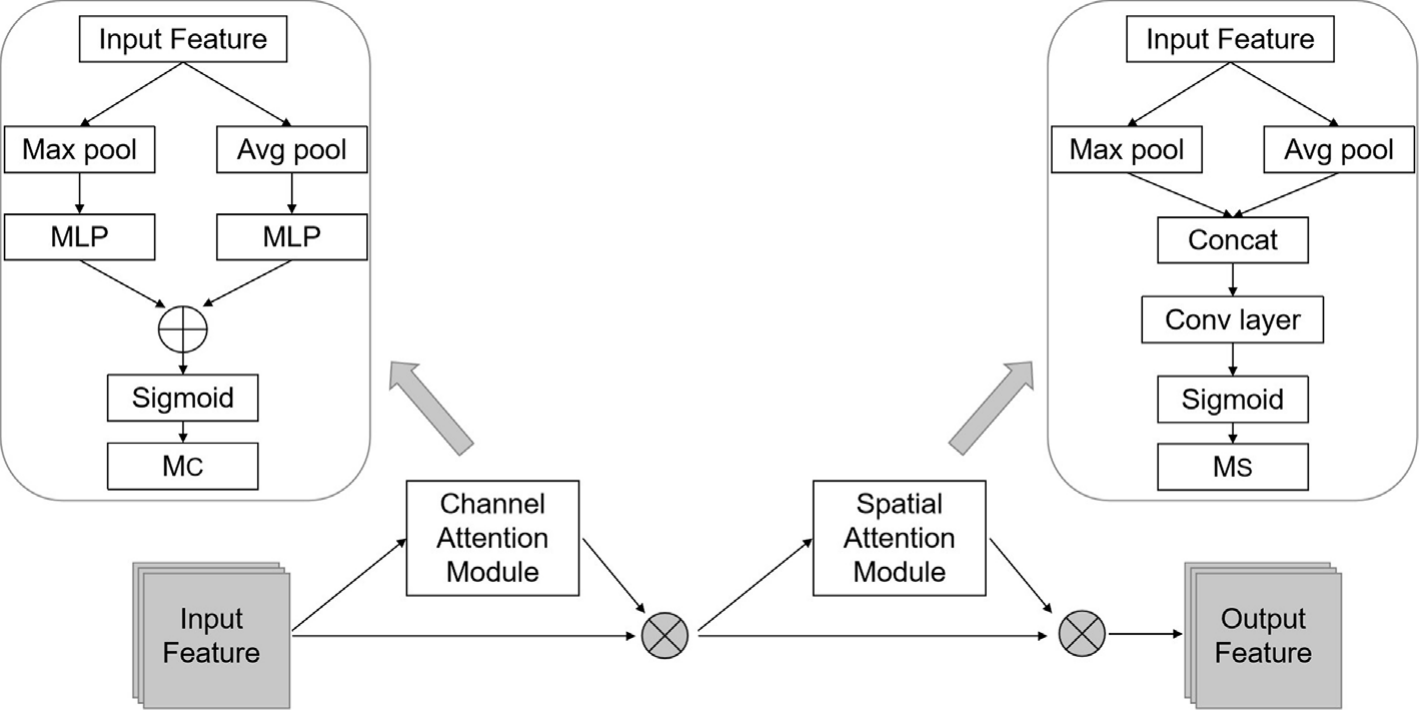
Detailed structure of the Convolutional Block Attention Module (CBAM) module.

In particular, the Denseblock is composed of 12 dense layers, where the function of the dense layer is shown in **Figure 5**. The batch normalization layer solves the problems of gradient disappearance and gradient explosion through data normalization. The ReLU layer is used to add nonlinear factors for a better expression ability, which is easier to train and often achieves better performance against alternatives (e.g., sigmoid, hyperbolic tangent). The dropout layer, a typical regularization technique for neural network models, can effectively reduce branches and avoid overfitting.

The Transition layer is placed between the dense block and the CBAM module. It consists of BN, ReLU, 1*1*1 Conv layer, and 2*2*2 average pooling layer. Its role is to reduce the dimension of each Dense Block output channel. There is a parameter reduction in the Transition Layer, which reduces the output to the original reduction times, where the default parameter of 0.5 is adopted in this article. The CBAM module is one type of attention mechanism module that combines space and channels. Compared against SE-Net, it is able to increase the attention mechanism of attention space and also achieve better results.

The channel attention mechanism is indeed similar to SE-Net, except that in addition to using the Avg pool when compressing the spatial dimensions of the Feature map, the Max pool is also added. When gradient back-propagation is performed, gradient feedback is provided where the feature map responds most to make the information more comprehensive. MLP is a two-level fully connected layer. The channel dimension is first reduced to 1/16 of the original and then raised back to the original dimension. It adds non-linearity and better fits the complex correlation between channels. The intermediate feature requires the ReLU Activate function to process. The mathematical expression of this process is given in Eq (1):

(1) $$M_{C}(F)=\sigma \;\left(MLP\left(AvgPool(F)\right)+MLP\left(MaxPool(F)\right)\right)$$

The spatial attention mechanism makes the network respond more to important parts of the Feature map at the spatial level. In this approach, a Global max pooling and Global average pooling are first done based on the feature channel, and then the concatenation operation is done. Then after a convolution operation, the dimension is reduced to 1 channel, which is followed by a Sigmoid operation to generate the Spatial attention feature. The mathematical expression for this process is given in Eq (2):

(2) $$M_{S}(F)=\sigma \left(f^{7\times 7}([AvgPool(F);MaxPool(F)])\right)$$

### C-DenseNet Variants

In addition to C-DenseNet, its variants are also proposed and tested. First, the so-called C-DenseNet-IN is considered, where the CBAM module is added in the dense block. In addition, the effect of the number of channels of each Dense Block is also tested, where C-DenseNet-121 (C-DenseNet equivalently), C-DenseNet-169, and C-DenseNet-201 are considered. The overall structure design is shown in **Table 3**, where k represents the growth rate. Modules are finally connected in a Concatenate way, so each time a module passes, the feature dimension of the next layer increases by k. A larger k means the smoother information in the network and, therefore, a stronger network. However, this is at the expense of increasing network size and calculation. k = 32 is chosen in this article.

**Table 3:** Detailed structure of C-DenseNet variants.

Layers	Output Size	C-DenseNet-121(k=32)	C-DenseNet-169(k=32)	C-DenseNet-201(k=32)
Convolution	640 × 640	7 × 7 conv,stride2		
Pooling	320 × 320	3 × 3max pool,stride2		
Dense Block 1	320 × 320	$$\left(\begin{array}{c} 1\times 1\text{ conv}\\ 3\times 3\text{ conv} \end{array}\right)\times 6$$		
Transition Layer 1	320 × 320	1 × 1 conv		
	160 × 160	2 × 2 average pool		
CBAM Layer 1	160 × 160	scale × 1		
Dense Block 2	160 × 160	$$\left(\begin{array}{c} 1\times 1\text{ conv}\\ 3\times 3\text{ conv} \end{array}\right)\times 12$$		
Transition Layer 2	160 × 160	1 × 1 conv		
	80 × 80	2 × 2 average pool		
CBAM Layer 2	80 × 80	scale × 1		
Dense Block 3	80 × 80	$$\left(\begin{array}{c} 1\times 1\text{ conv}\\ 3\times 3\text{ conv} \end{array}\right)\times 24$$	$$\left(\begin{array}{c} 1\times 1\text{ conv}\\ 3\times 3\text{ conv} \end{array}\right)\times 32$$	$$\left(\begin{array}{c} 1\times 1\text{ conv}\\ 3\times 3\text{ conv} \end{array}\right)\times 48$$
Transition Layer3	80 × 80	1 × 1 conv		
	40 × 40	2 × 2 average pool		
CBAM Layer 3	40 × 40	1 × 1scale		
Dense Block 4	40 × 40	$$\left(\begin{array}{c} 1\times 1\text{ conv}\\ 3\times 3\text{ conv} \end{array}\right)\times 16$$	$$\left(\begin{array}{c} 1\times 1\text{ conv}\\ 3\times 3\text{ conv} \end{array}\right)\times 32$$	$$\left(\begin{array}{c} 1\times 1\text{ conv}\\ 3\times 3\text{ conv} \end{array}\right)\times 32$$
Classification Layer	1 × 1	40 × 40 average pooling		
		fully-connected, softmax		

### Attention Visualization of Different Models

In order to visually analyze the changes brought by the addition of the attention module, this paper uses Grad-CAM++ (Chattopadhyay et al., 2017) to visualize the features of the wheat stripe rust leaf test set. Grad-CAM is an effective feature visualization method. This approach mainly uses the gradient of the target class to obtain the weight of the feature map, and then performs weighted summation to obtain the attention heat map. For the sack of completeness, Grad-CAM++ algorithm is briefly introduced. First, the weights are calculated by the global average of the gradient. Compared to Grad-CAM, Grad-CAM++ adds ReLU and weight gradients $\alpha_{ij}^{kc}$. We define the weight of the category c corresponding to the kth feature map in Grad-CAM as $w_{k}^{c}$ which is given by Eq (3):

(3) $$w_{k}^{c}=\sum_{i}\sum_{j}\alpha_{ij}^{kc}.relu\frac{\partial y^{c}}{\partial \;A_{ij}^{k}}$$

where *y^c^* is the gradient of the score for class c, $A_{ij}^{k}$ is the pixel value at the (i, j) position in the kth feature map. Then the weights of all the categories corresponding to the feature maps are obtained and weighted summation is performed, so that the final heat map can be obtained. The summation formula is shown in Eq(4):

(4) $$Y^{C}=\sum_{k}w_{k}^{c}.\sum_{i}\sum_{j}A_{ij}^{k}$$

## Results

### Model Training

To validate the performance of different models, 80% of the images in each category are randomly selected as training samples and the remaining 20% are utilized as validation samples. All the deep learning networks in this study were implemented by using a Python tool in the Pytorch framework. The computations were run on an Intel Xeon E5-2618L 2.3GHz PC with an NVIDIA GeForce GTX 2080 Ti graphics processing unit (GPU).

The basic parameters of C-DenseNet are shown in **Table 4**. In addition, the standard cross-entropy is utilized as the loss function in model training. To better address the problem of over-fitting and gradient vanishing, L2 regularization is exploited and the initial learning rate is set to be 0.01. In pursuit of a faster training speed, the strategy of ‘‘SGD+momentum’’ is utilized as the optimization algorithm. Weight-decay and momentum are set to be 1e-4 and 0.9, respectively.

**Table 4:** Basic parameters of C-DenseNet.

Hyper-parameters	Value
Image size/Batch size	(640, 640,3)/8
Epochs	50
Ratio/Kernel_size of the CBAM module	16/7

### Performance of C-DenseNet

**Figure 6:**
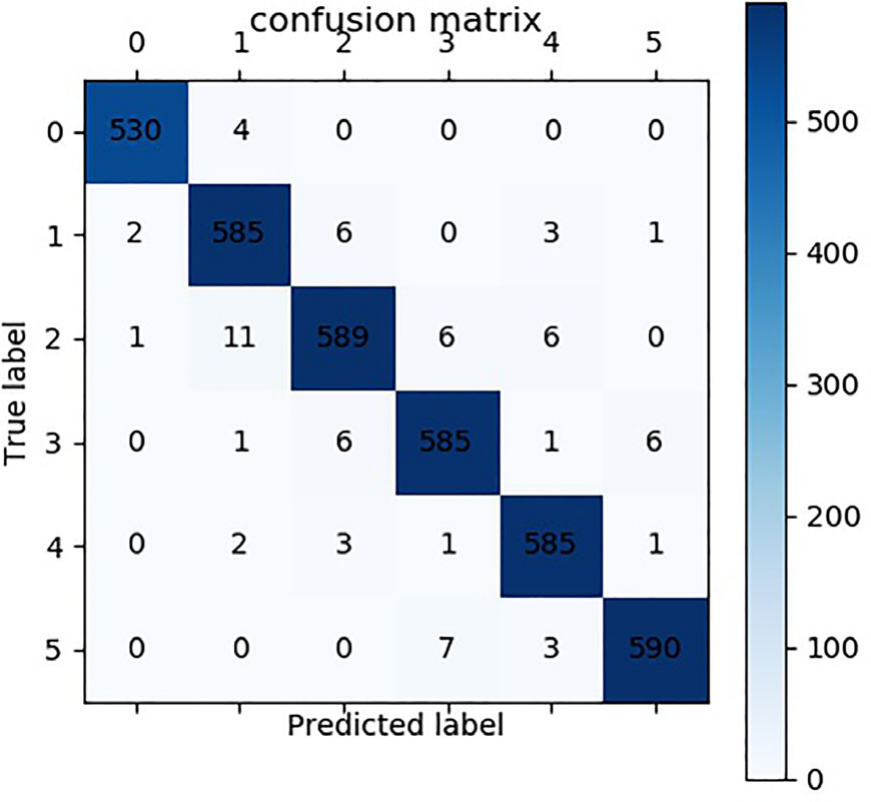
Confusion matrix of the proposed C-DenseNet.

Upon training the models, the hold-out 20% of the image dataset is used to evaluate the proposed C-DenseNet network against the classical DenseNet (Huang et al., 2017) and ResNet (Chang et al., 2018). A number of evaluation metrics are adopted in this work including accuracy, precision, recall and F1-score. In addition, confusion matrix is also used for the proposed C-DenseNet, which is shown in **Figure 6**. From the confusion matrix, it can be seen that the misclassification mainly occurs between levels 1 and 2, and between levels 3 and 5. This is mainly because the differences between them are very tiny and subtle, and, therefore, a few misjudgments are within the tolerable ranges. The sample pictures that were predicted incorrectly and accurately by the model are listed in a table to visually verify the performance of the model, as shown in **Table 5**.

**Table 5:**
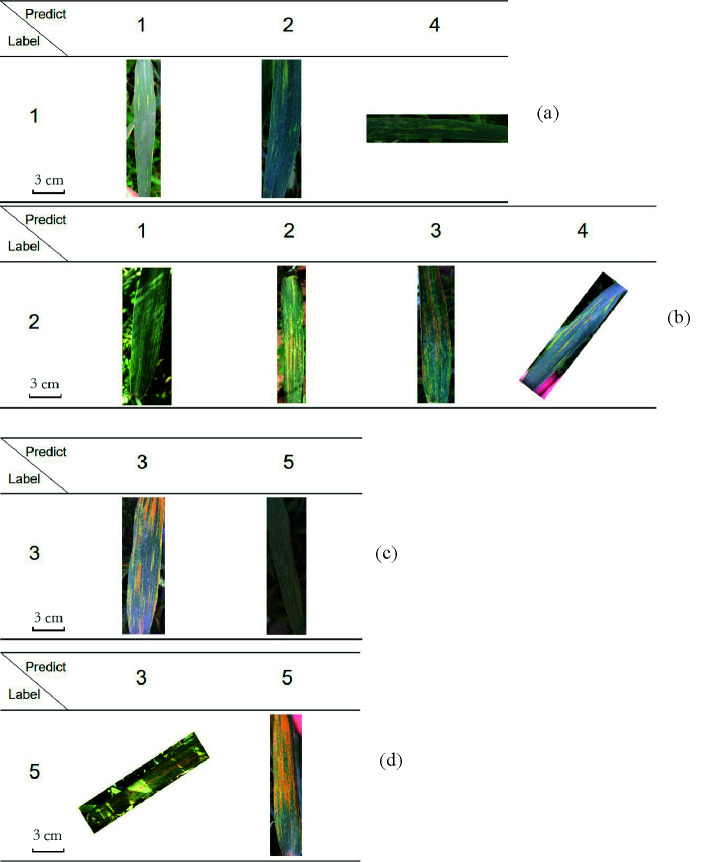
Some example wheat leaves which are predicted by this method correctly and incorrectly. (a), (b), (c), and (d) are for label 1, 2, 3, 5, respectively.

The performance in term of Accuracy, Precision, Recall and F-1 score for the proposed C-DenseNet, DenseNet, and ResNet is presented in **Table 6**. It is shown that both C-DenseNet and DenseNet significantly outperform the ResNet for all evaluation metrics. In addition, the proposed C-DenseNet also outperforms the DenseNet for all evaluation metrics, particularly with an improvement of 6% in accuracy. This result proves the effectiveness of the attention mechanism in wheat stripe rust disease grading.

**Table 6:** Performance comparison for the proposed C-DenseNet against the classical DenseNet, and ResNet.

Network	Accuracy	Precision	Recall	F1-score
ResNet	0.7343	0.7272	0.7276	0.7197
DenseNet	0.9253	0.9254	0.9253	0.9248
C-DenseNet	**0.9790**	**0.9799**	**0.9799**	**0.9799**

Each result is reported in the form of mean deviation. The ones in bold denote the best performance.

### Performance of C-DenseNet Variants

In the section, the performance of C-DenseNet variants is assessed against C-DenseNet by using cross-validation. Similarly, evaluation metrics including accuracy, precision, recall and F1-score are used and the results are shown in **Tables 7** and **8**. It can be seen from **Table 7** that the effect of placing the CBAM module in or outside the denseblock is neglectable, which indicate that the attention mechanism has been applied in multiple places in DenseNet effectively. It follows from **Table 8** that increasing the number of channels does not effectively improve the recognition accuracy, but instead increases the training cost.

**Table 7:** Performance comparison of C-DenseNet and C-DenseNet-IN.

Network	Accuracy	Precision	Recall	F1-score
C-DenseNet-IN	0.9702	0.9703	0.9703	0.9703
C-DenseNet	**0.9790**	**0.9799**	**0.9799**	**0.9799**

Each result is reported in the form of mean deviation. The ones in bold denote the best performance.

**Table 8:** Performance comparison against C-DenseNet-169 and C-DenseNet-201.

Network	Accuracy	Precision	Recall	F1-score
C-DenseNet-121	**0.9790**	**0.9799**	**0.9799**	**0.9799**
C-DenseNet-169	0.9439	0.9439	0.9439	0.9440s
C-DenseNet-201	0.9652	0.9600	0.9598	0.9598

The bold data denotes the one with the best performance.

### Network Attention Visualization

In order to investigate why the proposed C-DenseNet outperform other approaches, GradCAM++ is adopted to visualize the classification results. In particular, randomly selected examples in grade 2, 3 and 5 are tested, where the attention heat map are displayed in **Table 9**. It can be seen that C-DenseNet and C-DenseNet -IN, embedding CBAM attention mechanism, are able to pay more attention to the key areas in inferring the strip rust disease infection grade, while ResNet and DenseNet perform slightly worse.

**Table 9:**
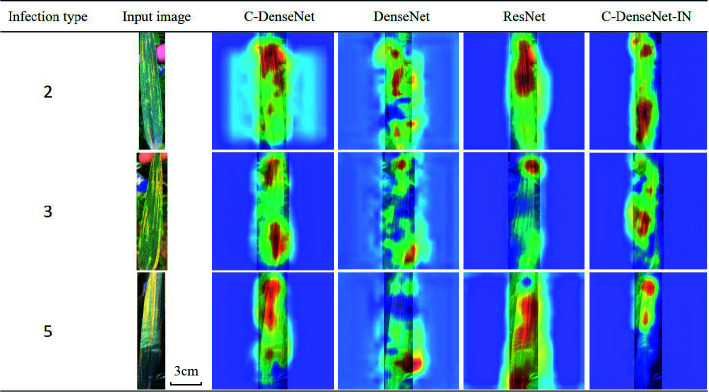
Attention heat maps of C-DenseNet, DenseNet, ResNet, and C-DenseNet networks for wheat leaves infected with grade 2, 3, and 5 level of stripe rust disease.

## Discussion

Wheat stripe rust is one of the most serious wheat diseases worldwide (88% of the world’s wheat production), which is extremely destructive in the main wheat production areas in China (Wan et al., 2004; Wellings, 2011; Ma et al., 2013). Growing disease-resistant cultivars is the most effective, economical, and environmentally sound approach to control wheat stripe rust (Chen, 2005; Dodds and Rathjen, 2010). In disease-resistant cultivar breeding, it is of great importance to assess the disease severity in their growing lifecycle. The traditional methods for disease grading rely on manual inspection, which is labor-intensive, inefficient, and subjective. Therefore, there is a clearly urgent need to develop more effective and efficient wheat strip rust disease grading algorithms, which can automatically classify wheat leaves into different categories of interest.

In the past few years, the performance of CNNs in target recognition and image classification has improved significantly (Schmidhuber, 2015; He et al., 2016). Although the recognition of plant diseases using images can be achieved (Mohanty et al., 2016; Ramcharan et al., 2017), there are still some problems to be addressed. On the one hand, most of the images used in the research are taken under a controlled environment. As a result, the trained model results in poor performance in complex field conditions. On the other hand, there is little research on wheat stripe rust disease grading. The differences between different grades of wheat leaves are usually tiny and subtle, bringing significant challenges for image classification. Therefore, this study develops deep learning based wheat strip rust disease grading algorithms by using color images taken by mobile devices.

In this study, we proposed C-DenseNet architecture for wheat stripe rust grading tasks. In C-DenseNet, the CBAM module is set between dense blocks of DenseNet. In dense blocks, each layer is feed-forward connected to all other layers, which allows new features to be extracted based on the features of the previous layer. However, feature redundancy in feature fusion is a major problem. Due to the advantages of useful automatic feature learning in the CBAM module, C-DenseNet can suppress redundant features, thereby mitigating the adverse impact of feature redundancy to a certain extent, and improving the performance of infective level grading tasks.

According to the experimental results in Section 3.2, the performance of C-DenseNet is better (i.e., 6% improvement in term of accuracy) than DenseNet in wheat stripe rust disease grading, confirming the advantages of the CBAM module in DenseNet. Besides, as shown in **Table 6**, the proposed C-DenseNet is significantly superior to ResNet (0.7343). In addition, it follows from **Table 7** that the advantages of placing the CBAM module in dense blocks (0.9702) are not as significant as placing CBAM module between dense blocks. In the process of exploring new features, the CBAM module in the dense block also learns weights of the features, which may cause overfitting, and the CBAM module between dense blocks can enhance useful features and suppress features that are not conducive to classification. This may lead to greater improvements in C-DenseNet performance. On the other hand, increasing the channel of the Dense Block does not effectively improve the hierarchical performance of the network, but instead increases network complexity and may also lead to overfitting.

## Conclusion and Future Work

This study aims to develop wheat strip rust disease grading algorithms in support of efficiently breeding disease-resistant wheat varieties for its sustainable management. To this end, the challenge is first formulated as a fine-grained image classification problem, then C-DenseNet is proposed by embedding Convolutional Block Attention Module (CBAM) in the classical densely connected convolutional network (DenseNet) to achieve adaptive calibration of feature channels and space. A wheat stripe rust grading dataset (WSRgrading dataset) is collected in field conditions with various mobile devices, which contain a total of 5,242 wheat leaves with 6 levels of wheat stripe rust infection. Extensively comparative experiments including model visualization are conducted to assess the performance of the proposed C-DenseNet and its variants against the classical DenseNet and ResNet. It is shown that the proposed C-DenseNet outperforms the classical DenseNet and ResNet, and, therefore, is suitable for wheat stripe rust disease grading in field conditions.

However, there is still much room for further improvement. (i) It would be of great practical significance to automatically isolate the leaves of interest under complex backgrounds in field conditions, eliminating the need for manual extraction. In addition, the data was taken in a wheat stripe rust breeding test field and, therefore, there are few pictures of other diseases or multiple diseases on one leaf. (ii) So the task of identifying multiple diseases of wheat was not taken into account in this study, and the proposed network is only applicable to grading of wheat stripe rust infection types.

## Data Availability Statement

The raw data supporting the conclusions of this article are available at https://github.com/xingyu960/C-DenseNet-for-wheat-stripe-rust-.

## Author Contributions

ZM designed and performed the experiment, selected algorithm, analyzed data, and wrote the manuscript. XZ trained algorithms and analyzed data. JS analyzed data and wrote the manuscript. DH collected data and monitored data analysis. BS conceived the study and participated in its design. All authors contributed to the article and approved the submitted version.

## Funding

This work was funded by the Fundamental Research Funds for the Central Universities (No.2452019028).

## Conflict of Interest

The authors declare that the research was conducted in the absence of any commercial or financial relationships that could be construed as a potential conflict of interest.
